# Factors influencing thyroidectomy complications

**DOI:** 10.1590/S1808-86942012000300012

**Published:** 2015-10-14

**Authors:** Miguel Ernandes Neto, José Vicente Tagliarini, Bárbara Estefania López, Carlos Roberto Padovani, Mariângela de Alencar Marques, Emanuel Cellice Castilho, Gláucia Maria Ferreira da Silva Mazeto

**Affiliations:** a Nephrologist (Private Practice).; b PhD (Assistant Professor - Head and Neck Surgery Program - Medical School of Botucatu; Unesp).; c MD. Public Health Specialist (Sanitarian, Botucatu University Hospital; Unesp).; d PhD (Assistant Professor – Department of Biostatstics – Biosciences Institute - Unesp).; e Senior Associate Professor (Department of Pathology – Medical School of Botucatu, Unesp).; f PhD (Physician at the Department of Ophthalmology and Otorhinolaryngology – Medical School of Botucatu, Unesp).; g PhD (Assistant Professor – Department of Internal Medicine – Medical School of Botucatu - Unesp). Hospital das Clínicas, Faculdade de Medicina de Botucatu; Universidade Estadual Paulista “Júlio de Mesquita Filho”.

**Keywords:** postoperative complications, postoperative period, thyroidectomy

## Abstract

The postoperative outcome of thyroidectomies is related to factors concerning the patient, the thyroid disease, and the surgeon.

**Objectives:**

To analyze a clinic's experience with thyroidectomy complications. Study design: historical cross-sectional cohort study.

**Materials and Methods:**

We reviewed the charts from 228 patients submitted to thyroidectomy, between 1991 and 2004. Transient, permanent and total complications as well as persistence and recurrence of the basal disease were studied in relation to clinical and laboratory factors.

**Results:**

Total complications occurred in 34.65%, transient complications in 18.86% (9.21% had hypocalcemia, 0.44% had vocal cord paralysis), associated with the first postoperative years and pressure complaints, and permanent complications in 17.98% (8.77%: hypoparathyroidism; 1.75%: vocal cord paralysis), associated with malignancy and more radical surgeries. The thyroid disease persisted in 17.98% of the cases, associated with age and recurrence in 10.96%, associated with the first operative years, benign diseases and less radical surgeries.

**Conclusion:**

The complications were associated with pressure complaints, shorter complaining period, malignancy and more radical surgeries. The recurrence was associated with the first operative years, non-neoplastic thyroid diseases and less radical surgeries. The persistence of disease was associated with older age.

## INTRODUCTION

Postoperative results from thyroid surgeries are usually related to patient condition, the thyroid disease, surgeon's experience and type of surgery^1^, ^2^. Thus, the percentages of undesirable results in thyroidectomy surgeries may vary considerably, considering baseline disease persistence or recurrence and postoperative complications^3^.

Thyroidectomy complications may be divided into transient or permanent. The transient may vary from severe, life threatening ones, all the way to mild and meaningless events. Permanent complications, which prevalence is variable, represent the main concern of those who treat thyroid diseases surgically. Despite its importance, the risk factors associated with post-thyroidectomy complications are not enough analyzed^4^.

The goal of the present study was to assess the experience of a clinic with thyroidectomy surgeries, analyzing the presence of undesirable results and the factors associated with them.

## MATERIALS AND METHODS

This study was approved by the Ethics in Research Committee of the institution (protocol 3625-2010). In order to evaluate the clinic's experience with this type of surgery, we assessed the medical charts from 228 patients submitted to thyroidectomy between 1991 and 2004. Our sample was made up of 209 women and 19 men, with ages between 8 and 78 years (mean ± Standard Deviation = 47.9 ± 14.8 years), who were followed up after surgery for a mean time of 40.3 months (Table 1). Clinical-laboratorial data from the patients were plotted and we considered the transient, permanent and total complications, besides the persistence and recurrence of the baseline thyroid disease, vis-à-vis the following parameters: gender; age (in years); clinical duration (in months); compressive complaints and their lack thereof; smoking; baseline thyroid status and status upon surgery (hyper, hypo or normal thyroid function); tests proving compression or showing a diving goiter; time when the surgery was carried out (between 1991 and 1997 or between 1998 and 2004); anesthetic risk (ASA 1 or 2); reason for the surgical indication (diving goiter, compression complaints, compression proven by tests, possible cancer, cancer or others); type of surgery carried out (lumpectomy/lobe-isthmectomy/lobectomy, subtotal or quasi-total/ total thyroidectomy); surgery in two procedures or not; it being a second operation or not; post-operative final diagnosis (colloid goiter, adenoma, malignant neoplasia or other diagnoses); final postoperative diagnosis of malignant neoplasia or not.

**Table 1:** Descriptive measures of some clinical-laboratorial parameters of patients submitted to thyroidectomy.

Parity									
Descriptive measure	Age (years)	Disease time (months)	Total # of FNA	2h capture (%)	6h capture (%)	Time to recurrence (months)	PO time for unknown destination (months)	Time span between coming to the clinic and surgery (months)	Follow up time (monts)
Number of samples	200	207	228	147	127	28	52	214	200
Minimum value	8.0	1.0	1.0	0.8	0.9	2.0	1.0	1.0	1.0
Median	49.0	36.0	3.0	4.3	7.1	33.0	7.5	28.0	23.0
Maximum value	78.0	756.0	9.0	40.0	42.6	111.0	110.0	338.0	144.0
Mean	47.9	79.4	3.1	5.7	9.1	42.6	24.1	47.6	40.3
Standard deviation	14.8	127.4	1.4	5.2	6.4	28.7	31.1	57.1	39.1

Recurrence was defined as the presence of baseline disease after it having been credibly resolved in the post-op. Persistence was defined as a lack of proven resolution of the baseline disease in the postoperative.

Data was plotted in the Excel^®^ spreadsheet, and later analyzed. In order to study the quantitative variables among themselves, we used association measures by means of the Pearson's correlation coefficient. The analysis of the quantitative variables in relation to the qualitative ones was carried out with the help of the Mann Whitney non-parametric test (for two groups). In order to study the qualitative data we used the Goodman test. The significance level adopted was 5 %.

Because it is a relatively small sample, the study must be continued in order to better assess the experience and the development of the clinic.

## RESULTS

Of all the patients studied, 134 (58.77%) were baseline with normal thyroid functions, while 50 (21.93%) were in hypo and 35 (15.35%) were hyperthyroidism. At the time of surgery, 148 (64.91%) had normal thyroid function, while 52 (22.81%) were in hyper and 19 (8.33%) were in hypothyroidism. Before surgery, 160 patients (70.18%) reported a past of goiter, 127 (55.70%) had compression complaints and 105 (46.05%) had a solitary or dominant goiter. Of the 199 patients who were submitted to tests to investigate compression, 75 (37.69%) really had it, while of the 222 in whom a diving goiter was investigated, 78 (35.14%) really had it. Other clinical-laboratorial findings from these patients can be seen on Table 1.

Surgery was indicated for the following reasons: suspected neoplasia in 72 (31.58%), compression confirmed in 55 (24.12%), cancer in 35 (15.35%), diving goiter (asymptomatic) in 30 (13.16%), compressive complaints in 23 (10.09%) and other causes in 13 cases (5.7%). Surgical risk was ASA 1 in 66 (28.95%), and ASA 2 in 125 patients (54.82%). The surgeries performed were partial (lumpectomy/lobe-isthmectomy/lobectomy) in 127 cases (55.7%) and total (quasi-total/total thyroidectomy) in 66 (28.95%). Subtotal thyroidectomy was done in 30 patients (13.16%).

The final postoperative diagnoses were: multinodular colloid goiter in 110 (48.25%), papilliferous carcinoma in 42 (18.42%), adenoma in 39 (17.11%), follicular carcinoma in 11 (4.82%), Hashimoto's thyroiditis in nine (3.95%), follicular hyperplasia in four (1.75%), Hürthle carcinoma in 2 (0.88%) and medullary carcinoma in one (0.44%) of the cases. In other words, the final diagnosis of malignant neoplasia was found in about 25% of the patients. It was not possible to establish the final postoperative diagnosis in 10 cases, because of missing documentation.

There were transient complications in 43 patients (18.86%), that is: hypocalcemia in 21 (9.21%), hoarseness in 15 (6.58%), vocal cord transient paralysis in one (0.44%); and others (hematoma, infection) in 11 cases (4.82%). The percentage of patients with them was higher in the group operated between 1998-2004 (22.5%) when compared to those submitted to surgery between 1991-1997 (11.1%; *p <* 0.05). Of the cases with transient complications, a higher percentage had compressive complaints (65.1% compared to 34.9%; *p* < 0.05) before surgery. All the other parameters investigated did not prove to be statistically significant among the patients with and without complications.

**Figure 1:**
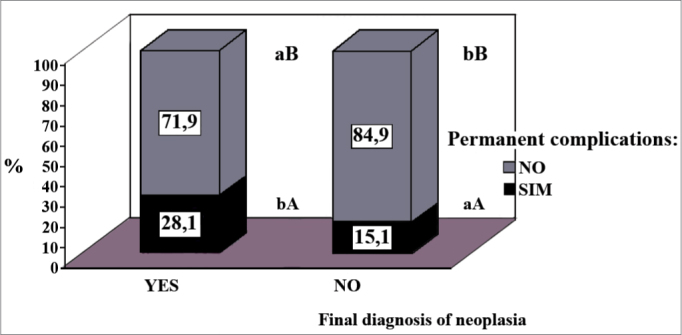
Percentage distribution of the thyroidectomized patients as to a final diagnosis of malignant neoplasia, associated with the occurrence or not of definitive surgical complications. The comparison within each group with or without malignant neoplasia, it is represented by upper case letters and, the one between groups is by lower case letters [Goodman test; b > a, B > A (*p* < 0.05)].

**Figure 2:**
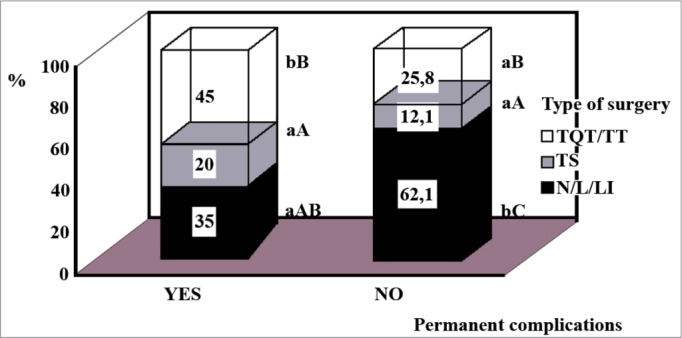
Percentage distribution of thyroidectomized patients, as to the definitive surgical complications, in relation to the type of surgery done (N/LI/L: lumpectomy/lobe-isthmectomy/lobectomy; ST: subtotal thyroidectomy; QTT/TT: quasi-total /total thyroidectomy). Comparison within each group, with or without complications, is represented by the upper case letters; and the one between the groups is in lower case ones [Goodman test; b > a, C > B > A (*p* < 0.05)].

Permanent complications were seen in 41 patients (17.98%): hypothyroidism in 22 (9.65%), hypoparathyroidism in 20 (8.77%), unilateral vocal fold paralysis in four (1.75%) and hoarseness in one patient (0.44%). The percentages of patients who had them were higher in the groups with a final diagnosis of malignant neoplasia (Figure 1) and in the one submitted to quasi-total/total thyroidectomy (Figure 2). History duration was shorter in the group with 22.0 ± 180.0 months when compared to the one without definitive complications (46.0 ± 378.0 months; *p* < 0.05). The other parameters analyzed did not show statistical significance.

Total complications, that is, permanent or transient, happened to 79 patients (34.65%). The percentage of patients who had them was higher in the groups with a final diagnosis of malignant neoplasia (49.1%) when compared to those without (29.9%; *p* < 0.05). By analyzing the total number of patients with complications, we noticed a higher percentage of cases with than without compression complaints (62% compared with 38%; *p* < 0.05). None of the other parameters studied showed statistically significant differences between the patients with and without total complications.

Baseline disease recurrence was seen in 25 patients (10.96%). The percentage of patients with it was higher in the group operated between 1991-1997 (26.2%) when compared to 1998-2004 (6.8%; *p* < 0.05) and in the group of patients without a final diagnosis of malignant neoplasia (17.6%) when compared with those with it (1.8%; *p* < 0.05). The percentage of patients submitted to lumpectomy/lobectomy/lobe-isthmectomy was higher in the recurrence group (88.5%), when compared to the one which did not relapse (49.1 %; *p* < 0.05); and the percentage of patients submitted to TQT/TT was higher in the group without recurrence (35.7%) than the one with it (3.8%; *p* < 0.05). Most of the patients without recurrence did not have preoperative exams showing compression (58.7% compared to 41.3%; *p* < 0.05) or a diving goiter (64.5% against 35.5%; *p* < 0.05). All the other parameters studied were not statistically different between the groups with and without recurrence.

Persistence of the baseline thyroid disease, which was the reason for the surgical indication, happened in 41 cases (17.98%). The patients with persistent disease were older (53.0 ± 30.0 years) than the ones without it (48.5 ± 34.0 years; *p* < 0.05). The other parameters analyzed did not show statistically significant differences between the groups with and without recurrence.

## DISCUSSION

Surgery may be a treatment option for benign thyroid diseases, and it is mandatory for their malignant counterparts. The type of surgery must be customized, depending especially on the diagnosis and surgeon experience. Ramirez et al.^5^ stated that complications associated with the thyroid surgery are directly proportional to the extension of the thyroidectomy and inversely proportional to the surgeon's experience.

Patients submitted to thyroid surgery are exposed to a number of complications, both in the early and in the late postoperative times, and the latter are usually permanent. Among those in the first group we have hypocalcemia, vocal fold paralysis, surgical wound infection and hematoma. Among those in the second group, the ones most frequently described are: hypoparathyroidism, permanent paralysis of the vocal folds and hypothyroidism. While hypocalcemia is considered the most important complication^6^, and the least desired; hypothyroidism is considered an expected result in total thyroidectomy cases^7^, although it may happen after less radical surgeries.

In the present study, there were transient complications in 18.86% of the cases. The literature reports a large variation in the rates of these complications, depending on type. In general, the prevalence varies between 5% and 27.5%^3^, ^6^, ^7^, ^8^. Thus, the results shown here do not point to much difference when compared to the experience of other clinics, national or international. There were a few cases of hematomas and postoperative infection. Reeve & Thompson^2^ reported that indeed, postoperative infections are the least common. Transient hypocalcemia, explained by the inflammation in the parathyroid glands – secondary to the surgical trauma, happened to 9.21% of the patients. In the literature there are rates varying from 2.43% in more conservative procedures, all the way to 35.29% in more extensive ones^4^, ^7^, ^8^, ^9^, ^10^.

Transient hoarseness, secondary to inflammation of the recurrent laryngeal nerve, happened in 6.57% and transient vocal fold paralysis happened to 0.43% of the patients. These figures are similar to the ones found by other authors. A Brazilian study, assessing patients operated because of atoxic multinodular goiter, relapsed or not, showed transient lesions of the laryngeal recurrent nerve from 2.7% to 5.0%, with the highest percentages in re-operated patients^9^. There is one international study which reported a transient paralysis of the recurrent laryngeal nerve in 1.8% of the cases^11^. Gonçalves Filho & Kowalski^6^, studying patients operated because of a differentiated carcinoma of the thyroid, showed 1.2% of such complication.

The percentage of patients with transient complications was higher in the group operated between 1998-2004 than those operated between 1991-1997. The reasons for such finding are still unclear, since the complications associated with the thyroid surgery are inversely proportional to the surgeon's experience^5^. In the group which had them, there was a striking percentage of patients with postoperative compressive complaints. Ríos et al.^12^ had 10% of transient hypoparathyroidism and 14% of transient dysphonia. These authors report that higher rates of complications in patients with compression complaints happen because of the greater technical difficulty during surgery^12^. We have to bear in mind that our clinic is in a University Hospital, where trainee physicians play an important role in the pre, intra and postoperative care of these patients, which certainly influences the rate of postoperative complications.

Permanent complications happened in 17.98% of the cases. Hypothyroidism happened in 9.65 % of the patients submitted to partial thyroidectomy, a complication of considerable prevalence in the literature^13^. However, we did not assess the association with autoimmune thyroiditis, it is known that it is frequent and causes hypothyroidism^14^. Insofar as total thyroidectomy procedures are concerned, hypothyroidism is expected, and not a complication^7^.

Permanent hypoparathyroidism happened to 8.77 % of the patients, most of whom were submitted to more radical surgeries. This complication is associated with the secondary removal, accidental or not, of the parathyroid glands. Other studies report rates between 2.5% and 8%^6^, ^7^, ^9^, ^11^, ^15^.

Permanent unilateral vocal fold paralysis, caused by irreversible lesion of the recurrent laryngeal nerve, happened in 1.75%. In the literature, we find references to permanent lesions, with rates varying between 0.2% and 5.0%^9^, ^11^, ^15^.

The percentages of patients with permanent complications were higher in the groups with a final diagnosis of malignant neoplasia, the one with individuals submitted to quasi-total/total thyroidectomy, and in that with a shorter history. This happened because patients diagnosed with malignant neoplasia are submitted to more radical surgeries, which could have more related complications, since complications associated with the thyroid surgery are directly proportional to the thyroidectomy extension^5^. According to the literature, permanent complications in patients submitted to total thyroidectomy to treat atoxic multinodular goiter were similar to those submitted to non-total thyroidectomy; nonetheless, lower than that of total thyroidectomy for relapsed goiter^9^.

The total rate of complications in the present study was 34.64%. This rate may be considered relatively high when compared to other studies: Zambudio et al.^4^, in 2004, reported 21% and Ríos et al.^12^ reported 24%, in patients with benign disease and symptoms of compression.

The percentage of patients who had total complications was higher in the groups diagnosed with malignant neoplasia and in the one which had complaints of compression before the surgery. Shen et al.^16^ reported 12% of complications in patients who complained of compression, and Rosato et al.^7^ found 5.7% of damage in the recurrent laryngeal nerve among patients with malignant neoplasia. We must stress that patients with malignant neoplasia and those with compression complaints are submitted to more radical surgeries, which would imply higher complication rates, although it is not widely accepted that all patients with thyroid cancer should be submitted to total thyroidectomy^9^.

We found relapses in 10.96% of the cases. Snook et al.^17^ showed rates of 0.32% and Bellantone et al.^15^had 0.0%. However, these authors investigated only total thyroidectomy procedures. The number of patients who had them was higher in the group operated between 1991-1997 (26.2%) when compared to that operated between 1998-2004 (6.8%) and in the one without a diagnosis of malignant neoplasia (17.6%). We believe this happened because of the longer follow up time of the first group. Moreover, those patients without a diagnosis of malignant neoplasia were submitted to more conservative procedures, enabling recurrences of benign thyroid diseases. The role of total thyroidectomy in benign thyroid disease still remains controversial in the literature, although minimizing the recurrence/reoperation rates^10^, because of higher complication rates associated to more radical surgeries^8^. The number of patients submitted to lumpectomy/lobectomy/lobe-isthmectomy was higher in the group with recurrences (88.5%) when compared to the group which did not relapse (49.1%). The percentage of patients submitted to quasi-total/total thyroidectomy was higher in the group without recurrences (35.7%) when compared to the one with it (3.8%). Snook et al.^17^ reported that total thyroidectomy is not the only safe procedure to treat benign thyroid goiter, but it is efficient and prevents relapses. According to Rosário et al.^18^, the extension of surgery is associated with lower rates of recurrence, distant metastasis and mortality in patients with malignant thyroid neoplasia submitted to bilateral thyroidectomy.

Baseline thyroid disease persisted in 17.98% of the patients. This seems to depend on the nature of the thyroid disease operated. Mazzaferri et al.^19^ showed a 25% persistence rate in 25% of the patients operated because of papilliferous carcinoma of the thyroid, while the persistence of hyperthyroidism in patients submitted to surgery should be less than10%-20%^20^. In the present study, the patients with persistent disease were older than those without it. Patient age is considered an important predicting factor of postoperative complications, but not vis-à-vis disease persistence in head and neck surgeries^21^.

## CONCLUSION

With our patients we had considerable rates of undesirable results after thyroidectomy. Of these, the most striking were postoperative complications, associated with compression, short history, malignant neoplasia and more radical surgeries. Baseline thyroid disease recurrence was associated with our first years of experience with this surgery, with non-neoplastic thyroid diseases and less radical surgeries. Nonetheless, disease persistence was associated with patient advanced-age.
